# Knowledge, attitude, and correlates of HIV post-exposure prophylaxis among medical students at three medical schools in China: a multi-center cross-sectional study

**DOI:** 10.3389/fpubh.2026.1875173

**Published:** 2026-07-06

**Authors:** Maoqi Xiong, Xiao He, Manqing He, Chao Zhang, Lina Duo, Jun Chen, Anjie Ren, Dan Pu

**Affiliations:** 1 Clinical Skills Training Center, West China Hospital, Sichuan University, Chengdu, China; 2 Department of Dermatology & Medical Aesthetics, Chengdu Seventh People’s Hospital, Chengdu, China; 3 Department of Medical Services Management, Peking University People’s Hospital, Beijing, China; 4 Education & Healthcare Research Center, Chengdu Gongyun Education & Management Research Institute, Chengdu, China

**Keywords:** China, healthcare students, HIV prevention education, medical curriculum, post-exposure prophylaxis literacy, risk communication, stigma

## Abstract

**Background:**

HIV post-exposure prophylaxis (PEP) is effective when initiated promptly and used correctly, but uptake remains limited partly because potential users and future providers may not recognize when or how PEP should be used. Medical students are relevant for assessment because they represent future clinicians and may also encounter occupational exposure during training. Evidence on PEP-specific knowledge and attitudes among Chinese medical students remains limited.

**Methods:**

We conducted a multi-center cross-sectional survey between March and August 2025 among 1,382 medical students at three medical schools in China. A structured 54-item online questionnaire assessed PEP knowledge, PEP attitudes, and HIV-related stigma. Knowledge and attitude scores were analyzed as continuous outcomes. Multivariable linear regression models were used to examine factors associated with knowledge and attitudes.

**Results:**

The mean PEP knowledge score was 12.1 (SD = 4.3) on a 0–20 scale, and the mean attitude score was 52.8 (SD = 8.6) on a 15–75 scale. Knowledge was highest for basic PEP definition, the 72-h initiation window, and the 28-day regimen, but lower for delaying PEP until a baseline HIV test result is available, the HIV testing window period, and cost-related access. In adjusted analyses, higher knowledge was associated with academic year (beta = 0.26), prior PEP training (beta = 0.22), formal HIV curriculum exposure (beta = 0.12), and attendance at Site 1 relative to Site 3 (all *p* < = 0.004). More favorable attitudes were associated with higher knowledge (beta = 0.28), prior PEP training (beta = 0.15), clinical rotation completion (beta = 0.09), female sex (beta = 0.07), and attendance at Site 1 relative to Site 3 (all *p* < = 0.022). Knowledge and attitude scores were positively correlated (*r* = 0.29, *p* < 0.001).

**Conclusion:**

In this large multi-site convenience sample, PEP knowledge was uneven and was weaker for applied clinical details than for general facts, whereas attitudes toward PEP were broadly favorable. Prior PEP-specific training was consistently associated with stronger scores. Because recruitment relied on institutional WeChat distribution rather than probability sampling, the findings should be interpreted as evidence from participating schools and should not be generalized directly to all medical students in China.

## 1 Introduction

Human immunodeficiency virus (HIV) remains a major global public health challenge, with an estimated 39 million people living with the infection worldwide. In China, national surveillance has documented a continuing shift toward sexual transmission, with heterosexual and male-to-male sexual contact accounting for the dominant share of newly reported infections and with young adults remaining an important population for prevention education (1, 2). Although the overall epidemic has been described as low prevalence at the population level, regional heterogeneity, delayed testing, and uneven access to prevention services continue to create public health challenges. Post-exposure prophylaxis (PEP) is a time-sensitive biomedical prevention strategy that involves a 28-day course of antiretroviral therapy initiated within 72 h of a potential exposure to HIV (3). When adhered to correctly, PEP substantially reduces the likelihood of HIV acquisition and has been recommended as a core component of comprehensive HIV prevention programs in international and Chinese guidelines (1, 3, 4). Despite its proven efficacy, the utilization of PEP remains suboptimal, often hindered by low awareness, limited knowledge of the regimen, and operational barriers related to access and adherence (5–7). Understanding knowledge gaps and attitudes toward PEP among populations who are both potential users and future providers is therefore a critical step in strengthening HIV prevention preparedness.

The relevance of PEP knowledge in China is underscored by the country’s evolving HIV prevention landscape and the growing emphasis on biomedical interventions within the national strategy (3, 4). Chinese guidelines have formally introduced both non-occupational post-exposure prophylaxis (nPEP) and occupational PEP protocols, alongside pre-exposure prophylaxis (PrEP), as part of the HIV prevention toolkit (4). However, awareness and uptake remain low among high-risk groups, including men who have sex with men, female sex workers, and people who use drugs (2, 8, 9). In this context, medical students represent a consequential population for study. These individuals will soon become frontline clinicians responsible for PEP provision, risk assessment, and patient education across diverse healthcare settings (5). At the same time, many medical students are young adults who may themselves be at risk of HIV exposure through sexual activity or occupational needlestick injuries, making them both potential users of PEP and future agents of its dissemination (10, 11, 42). A robust understanding of PEP indications, timing, regimen characteristics, and follow-up requirements is essential for appropriate referral, prescribing, and patient counseling (12, 13).

Despite the recognized importance of PEP and the central role that medical students will play in its delivery, no study has yet assessed PEP-specific knowledge and attitudes simultaneously across multiple medical schools in China. Existing evidence is largely derived from single-institution surveys or from studies that conflate PEP knowledge with general HIV knowledge, limiting the ability to describe curriculum-level and institutional-level variation (5, 10). Moreover, attitudes toward PEP, including perceived importance, willingness to recommend, and confidence in discussing the intervention with patients, have rarely been quantified alongside knowledge scores in Chinese medical student populations (14, 15). This gap is consequential because knowledge and attitudes may be related but distinct dimensions of preparedness; a cross-sectional association between them would not establish causality, but it may identify areas for educational attention. Given the increasing emphasis on biomedical HIV prevention in national policy (3, 4), an understanding of how well medical students at participating schools are prepared to discuss and navigate PEP services is needed. Therefore, this study aimed to assess PEP knowledge and attitudes among medical students at three medical schools in China and to examine demographic, educational, and institutional correlates of score variation.

## 2 Materials and methods

### 2.1 Study design and setting

This multi-center cross-sectional survey was conducted between March 3 and August 12, 2025, among medical students enrolled at three medical schools in China. Site 1 was a large comprehensive medical school in Southwest China with approximately 7,800 medical students and multiple tertiary teaching hospitals, including infectious disease and dermatology/STI outpatient services. Site 2 was a provincial medical university in Southwest China with approximately 6,300 medical students and affiliated general hospitals, where HIV prevention content was mainly delivered through infectious disease and public health courses. Site 3 was a large medical school in Central China with approximately 5,200 medical students and teaching-hospital placements, but no uniformly required PEP-specific competency assessment at the time of the survey. Across sites, HIV-related teaching was included in standard curricula, whereas dedicated PEP training, seminars, and clinical protocol exposure varied by department and academic year. The study was coordinated by the Chengdu Gongyun Education & Management Research Institute.

### 2.2 Participants and sampling

Eligible participants were currently enrolled medical students, at undergraduate or graduate level, aged 18 years or older at one of the three participating institutions. Responses were considered analytically valid if at least 80% of questionnaire items were completed and the submission time was at least 180 s. Responses with obvious response-pattern artifacts, defined *a priori* as identical selection of the same response category across all Likert items together with failure of at least one embedded attention-check item, were excluded from the primary dataset.

A convenience sampling strategy was employed. The online survey link was hosted on the Wenjuanxing platform^1^ and distributed through institutional WeChat groups used for class, grade, and student-affairs communication. Site coordinators and class advisors forwarded a standardized invitation, but no roster-based sampling frame was available. Participation was voluntary, and no financial compensation, academic credit, or other incentive was offered. Because recruitment occurred through group distribution rather than direct individual invitation, the number of students who saw the invitation could not be verified and a conventional response rate could not be calculated. In total, 1,527 submissions were logged; 61 were excluded for completion below 80%, 39 for response time below 180 s, 27 for patterned responding, and 18 for duplicate, ineligible, or technically corrupted records, leaving 1,382 analytically valid responses. The retained sample comprised 552 participants from Site 1, 443 from Site 2, and 387 from Site 3. The resulting sample was intended to provide stable estimates within the participating institutions and to support multivariable association analysis, but it was not designed to be statistically representative of all medical students in China.

### 2.3 Instruments

The structured questionnaire was developed by the research team on the basis of existing HIV knowledge, attitude, and practice instruments and World Health Organization guidance on HIV post-exposure prophylaxis. Because no fully validated Chinese instrument specifically measuring medical students’ HIV PEP knowledge and attitudes was available for the present study objectives, the instrument was study-specific. Initial items were drafted to cover guideline-based PEP timing, regimen duration, occupational and non-occupational indications, baseline and follow-up testing, access pathways, and counseling attitudes. The draft instrument underwent two rounds of expert review by three specialists in HIV prevention, infectious disease education, and survey design. Experts rated each substantive item for relevance and clarity on a four-point scale; the item-level content validity index ranged from 0.83 to 1.00, and the scale-level average content validity index was 0.94. The revised questionnaire was pilot-tested in 30 medical students who were not included in the final sample. Minor wording changes were made to simplify regimen-related and follow-up-testing items, and median completion time in the pilot was 8.6 min. The final instrument comprised 54 items distributed across seven components: informed consent (1 item), demographics (9 items), PEP knowledge (20 items), PEP attitudes (15 items), HIV-related stigma (5 items), attention checks (3 items), and a self-rated response honesty item (1 item). These procedures supported content relevance and internal consistency but did not constitute comprehensive psychometric validation.

*Demographics*: Nine items captured participant characteristics, including age, sex, academic year, institution, pre-enrollment residence, formal HIV/AIDS curriculum exposure, prior PEP-specific training or lectures, completion of at least one clinical rotation, and prior occupational HIV exposure experience.

*PEP knowledge*: Knowledge of HIV post-exposure prophylaxis was assessed using 20 items covering PEP definition, time window for initiation, recommended duration, standard regimen, efficacy, indications for occupational and non-occupational exposure, distinction from pre-exposure prophylaxis, side effects, follow-up testing, baseline testing procedures, service availability in China, cost, use during pregnancy, re-use restrictions, impact on the HIV testing window period, adherence requirements, and occupational exposure management. Each item was presented as a statement and participants indicated whether the statement was correct, incorrect, or unsure. Each correct answer was scored as 1 point, and incorrect or unsure responses were scored as 0, yielding a total knowledge score ranging from 0 to 20. Knowledge score was analyzed primarily as a continuous outcome. For descriptive interpretation only, scores were also grouped as low (0–9), intermediate (10–14), and high (15–20). Reliability of the dichotomous knowledge scale was evaluated using Kuder–Richardson Formula 20, and corrected item-total correlations were reviewed before final analysis.

*PEP Attitudes*: Attitudes toward HIV PEP were measured using 15 items rated on a five-point Likert scale ranging from 1 (strongly disagree) to 5 (strongly agree). The items assessed willingness to seek and recommend PEP, perceived importance of PEP as a prevention strategy, support for PEP integration into medical curricula, confidence in PEP knowledge and communication, stigma and moral concerns related to PEP provision, perceived barriers to PEP access, cost concerns, side-effect concerns, confidentiality concerns, and equitable provision regardless of exposure route. Seven items were reverse-coded to mitigate acquiescence bias. The total attitude score was computed as the sum of all 15 items after reverse-coding, yielding a possible range of 15 to 75, with higher scores reflecting more favorable attitudes toward PEP. The summed score was treated as a continuous outcome in all inferential analyses. For descriptive interpretation only, scores above the scale midpoint of 45 were considered more favorable than unfavorable overall. Because the attitude domain was specified *a priori*, confirmatory factor analysis was used as a pragmatic structure check rather than as definitive scale-development evidence. Internal consistency was assessed with Cronbach’s alpha. Corrected item-total correlations were also inspected, with values of 0.30 or greater considered acceptable for retention.

*HIV-Related Stigma*: HIV-related stigma was assessed using five items adapted from validated stigma scales for healthcare settings. Items addressed social distance, blame attribution, disclosure expectations, fear of providing care to people living with HIV, and endorsement of equal healthcare rights. Each item was rated on a five-point Likert scale, and two items were reverse-coded. The total stigma score ranged from 5 to 25, with higher scores indicating greater HIV-related stigma. The stigma scale was treated as a secondary contextual construct. It was described in its own results subsection and examined in correlation analyses, but it was not entered into the primary multivariable models because the *a priori* modeling aim was to evaluate demographic and educational correlates of knowledge and attitude.

*Attention Checks and Quality Control*: Three attention-check items were embedded at approximately the 25th, 50th, and 85th percentiles of the questionnaire to identify inattentive responding. Participants who failed two or more attention checks were retained in the primary analysis unless they also met exclusion criteria for patterned responding or insufficient completion, and they were examined in sensitivity analyses. Additional quality-control measures included single-use survey links within institutional distribution chains, platform-level blocking of duplicate submissions from the same device within the active survey session, a minimum response-time threshold of 180 s, and mandatory completion of all core knowledge and attitude items. The observed completion-time distribution in retained participants had a median of 9.1 min and an interquartile range of 6.8 to 11.9 min. A final item assessed self-rated response honesty on a five-point scale and was used only in sensitivity analyses.

### 2.4 Procedure

After study approval, the survey link and a standardized recruitment message were disseminated to target participants via WeChat. The message stated that the study was voluntary, anonymous, and unrelated to course grading or institutional evaluation. The landing page presented the study description, participation requirements, confidentiality assurances, and the online consent form. Participants indicated consent by selecting the corresponding option before proceeding to the questionnaire. No direct identifiers were collected in the exported analytic file. Completed responses were downloaded after survey closure and screened according to the predefined quality-control rules.

### 2.5 Statistical analysis

All statistical analyses were performed using SPSS (version 26.0; IBM Corp., Armonk, NY, USA) and R (version 4.3; R Foundation for Statistical Computing, Vienna, Austria). Categorical variables were summarized as frequencies and percentages. Continuous variables were summarized as mean ± standard deviation because both primary outcomes showed approximately symmetric distributions on inspection of histograms and Q-Q plots. Core outcome items were mandatory in the online questionnaire. Item-level missingness among retained cases was 0.0% for knowledge items, 0.0% for attitude items, 0.3% for stigma items, and 0.6% or less for demographic covariates. Complete-case analysis was used without imputation.

Bivariate analyses were conducted to examine associations between participant characteristics and the two primary outcomes, PEP knowledge score and PEP attitude score. Independent-samples *t-tests* or one-way analysis of variance were used for categorical predictors, and Pearson correlation coefficients were used for age and scale-score relationships. Cohen’s d was calculated for two-group comparisons, and partial eta-squared was calculated for omnibus ANOVA and ANCOVA models. Between-institution comparisons of knowledge and attitude scores were examined using analysis of covariance adjusted for age, sex, and academic year. This ANCOVA was used as a descriptive site-comparison model with a common minimal adjustment set, whereas the regression models were used for fuller multivariable association analysis.

Two separate multiple linear regression models were fitted to examine factors associated with PEP knowledge and PEP attitudes. Model construction was guided by the study’s conceptual framework and by variables plausibly related to educational exposure or clinical preparedness rather than by automated variable selection. For the knowledge model, prespecified predictors were age, sex, academic year, institution, prior PEP training, HIV curriculum exposure, and clinical rotation experience. For the attitude model, the same predictors were entered, with knowledge score added as a covariate because factual knowledge was considered conceptually proximal to attitude formation. Academic year was coded as an ordered six-level variable, and institution was dummy-coded with Site 3 as the reference group. Standardized beta coefficients with 95% confidence intervals were reported. Multicollinearity was assessed using variance inflation factors, with values above 5.0 considered problematic. Model diagnostics included inspection of residual plots, Q-Q plots, and Cook’s distance. As a robustness check, heteroscedasticity-consistent standard errors were estimated in parallel models; because the pattern of significance was unchanged, conventional model estimates are reported in the main text.

Sensitivity analyses were performed by excluding participants who failed two or more attention checks, participants with response times below 240 s, and participants who self-rated their response honesty as very untruthful or somewhat untruthful. Pairwise institutional comparisons following significant omnibus tests were evaluated with Bonferroni correction. A two-tailed alpha of 0.05 was applied throughout. Because the study was observational and exploratory with respect to several secondary comparisons, emphasis was placed on effect sizes, confidence intervals, and consistency of direction rather than on dichotomous interpretation of marginal *p-values* alone.

### 2.6 Ethics considerations

Ethical approval for the study was granted by the Ethics Committee of Chengdu Gongyun Education & Management Research Institute (approval number 25-017). The survey was anonymous, no direct identifiers were collected in the exported dataset, and electronic informed consent was obtained before questionnaire access.

## 3 Results

### 3.1 Sample characteristics

A total of 1,382 medical students from three Chinese medical schools were included in the analysis. Table 1 presents the demographic and educational characteristics of the sample. The mean age was 21.4 years (*SD* = 2.3), and 58.2% of participants were female (*n* = 805). Across the five undergraduate academic years and graduate level, the distribution was Year 1, 18.3% (*n* = 253); Year 2, 20.5% (*n* = 284); Year 3, 22.1% (*n* = 305); Year 4, 15.8% (*n* = 218); Year 5, 12.4% (*n* = 171); and graduate students, 10.9% (*n* = 151). Most participants reported an urban pre-enrollment residence (62.5%, *n* = 863), and 68.7% (*n* = 949) reported formal HIV/AIDS education in their curriculum. Only 23.4% (*n* = 323) reported prior PEP-specific training or lectures. Clinical rotations had been completed by 41.2% (*n* = 570), and 6.8% (*n* = 94) reported prior occupational HIV exposure. The median completion time among retained responses was 9.1 min (IQR 6.8–11.9).

**Table 1:** Participant characteristics by institution (*N* = 1,382).

Characteristic	Total (*N* = 1,382)	Site 1 (*n* = 552)	Site 2 (*n* = 443)	Site 3 (*n* = 387)	*p*-value
Age (years), M ± SD	21.4 ± 2.3	21.6 ± 2.2	21.3 ± 2.4	21.2 ± 2.3	0.18
Sex, *n* (%)					0.012
Female	805 (58.2)	335 (60.7)	248 (56.0)	222 (57.4)	
Male	577 (41.8)	217 (39.3)	195 (44.0)	165 (42.6)	
Academic year, *n* (%)					<0.001
Year 1	253 (18.3)	102 (18.5)	82 (18.5)	69 (17.8)	
Year 2	284 (20.5)	108 (19.6)	95 (21.4)	81 (20.9)	
Year 3	305 (22.1)	118 (21.4)	102 (23.0)	85 (22.0)	
Year 4	218 (15.8)	90 (16.3)	68 (15.3)	60 (15.5)	
Year 5	171 (12.4)	78 (14.1)	48 (10.8)	45 (11.6)	
Graduate	151 (10.9)	56 (10.1)	48 (10.8)	47 (12.1)	
Residence, *n* (%)					0.42
Urban	863 (62.5)	356 (64.5)	268 (60.5)	239 (61.8)	
Rural	519 (37.5)	196 (35.5)	175 (39.5)	148 (38.2)	
HIV curriculum, *n* (%)	949 (68.7)	392 (71.0)	298 (67.3)	259 (66.9)	0.15
PEP training, *n* (%)	323 (23.4)	155 (28.1)	95 (21.4)	73 (18.9)	<0.001
Clinical rotation, *n* (%)	570 (41.2)	265 (48.0)	172 (38.8)	133 (34.4)	<0.001
Occupational exposure, *n* (%)	94 (6.8)	42 (7.6)	31 (7.0)	21 (5.4)	0.41

M, mean; SD, standard deviation. *p*-values from chi-square tests (categorical) or one-way ANOVA (continuous).

Institutional composition differed on sex, academic year, prior PEP training, and clinical rotation completion. Because the survey used convenience recruitment through institutional communication groups, the sample should be interpreted as a large multi-site student sample rather than a population-representative cohort.

### 3.2 PEP knowledge

The overall mean PEP knowledge score was 12.1 (*SD* = 4.3; range: 0–20), corresponding to a mean correct-response proportion of 60.5%. For descriptive interpretation, 38.6% of participants (*n* = 533) scored in the high range (15–20), 34.8% (*n* = 481) in the intermediate range (10–14), and 26.6% (*n* = 368) in the low range (0–9). The 20-item knowledge scale showed acceptable internal consistency (KR-20 = 0.78), and corrected item-total correlations ranged from 0.24 to 0.58.

Item-level analysis (Table 2) showed marked variation across knowledge domains. The highest correct-response rates were observed for basic PEP definition (81.3%), the 72-h initiation window (76.8%), and the 28-day treatment duration (74.2%). Lower correct-response rates were observed for the impact of PEP on the HIV testing window period (22.4%), the item on delaying PEP until a baseline HIV test result is available (28.7%), effectiveness beyond 72 h (35.2%), and cost-related access in China (41.3%). Knowledge gaps therefore clustered in operational domains that require application rather than simple recall.

**Table 2:** PEP knowledge by item (*N* = 1,382).

Item	Content (abbreviated)	Correct answer	*n* (%) correct	Site 1 (%)	Site 2 (%)	Site 3 (%)
B-01	PEP definition	True	1,123 (81.3)	85.7	78.3	77.0
B-02	72-h time window	True	1,061 (76.8)	81.5	74.3	71.6
B-03	PEP effective beyond 72 h	False	486 (35.2)	40.2	32.1	30.2
B-04	28-day duration	True	1,025 (74.2)	80.8	70.2	68.0
B-05	Three-drug regimen	True	789 (57.1)	64.1	52.4	50.6
B-12	Follow-up testing schedule	True	618 (44.7)	50.2	41.3	39.3
B-13	Delay PEP until baseline HIV test result	False	397 (28.7)	33.9	25.7	23.5
B-18	Window period prolongation	True	310 (22.4)	26.8	19.9	17.8
B-19	Adherence requirement	False	912 (66.0)	71.7	63.2	59.4
B-20	Needlestick management	True	987 (71.4)	77.2	68.4	65.1

### 3.3 PEP attitudes

The mean PEP attitude score was 52.8 (*SD* = 8.6; range: 18–75), equivalent to a mean item score of 3.52 out of 5. The 15-item attitude scale showed good internal consistency (Cronbach’s alpha = 0.85), and corrected item-total correlations ranged from 0.36 to 0.69. Confirmatory factor analysis indicated acceptable, though not close, fit for a one-factor structure (*CFI* = 0.93, *RMSEA* = 0.061, 90% *CI* [0.055, 0.067], *SRMR* = 0.054). For descriptive interpretation, 65.4% of participants (*n* = 904) scored above 50, indicating an overall favorable orientation toward PEP on the summed scale.

Item-level attitude results are presented in Table 3. Participants expressed strongest agreement with the importance of PEP as an HIV prevention strategy (*M* = 4.02, *SD* = 0.71) and the need to include PEP in medical curricula (*M* = 3.95, *SD* = 0.78). Lower item means were observed for perceived accessibility of PEP in China (*M* = 2.56 after reverse-coding, *SD* = 0.97) and confidence in explaining PEP to patients (*M* = 2.73 after reverse-coding, *SD* = 0.95), suggesting that attitude was more positive toward the concept of PEP than toward practical readiness or service access (Table 3).

**Table 3:** PEP attitude by item (*N* = 1,382).

Item	Content (abbreviated)	Direction	M ± SD total	M ± SD site 1	M ± SD site 2	M ± SD site 3
C-01	Willingness to seek PEP	+	3.82 ± 0.88	3.95 ± 0.82	3.74 ± 0.90	3.70 ± 0.92
C-02	Willingness to recommend PEP	+	3.68 ± 0.92	3.80 ± 0.88	3.62 ± 0.94	3.55 ± 0.95
C-03	PEP importance	+	4.02 ± 0.71	4.10 ± 0.68	3.97 ± 0.73	3.95 ± 0.74
C-06	Confidence in explaining PEP	R	2.73 ± 0.95	2.88 ± 0.90	2.62 ± 0.98	2.60 ± 0.97
C-08	PEP accessibility in China	R	2.56 ± 0.97	2.68 ± 0.95	2.48 ± 0.98	2.45 ± 0.98
C-14	Moral judgment about PEP	R	3.45 ± 1.02	3.58 ± 0.98	3.38 ± 1.05	3.32 ± 1.04
C-15	Overall PEP value	+	3.95 ± 0.74	4.02 ± 0.70	3.90 ± 0.77	3.88 ± 0.76

Scale: 1 = strongly disagree to 5 = strongly agree. R = reverse-coded; values shown after recoding (higher = more favorable).

### 3.4 HIV-related stigma

The mean HIV-related stigma score was 12.9 (*SD* = 3.4; range: 5–25). The five-item stigma scale showed acceptable internal consistency for a brief contextual measure (Cronbach’s alpha = 0.70), with corrected item-total correlations ranging from 0.31 to 0.49. Stigma was higher among participants without prior PEP training (13.3 ± 3.5 vs. 11.8 ± 3.0; *t* = 6.67, *p* < 0.001; Cohen’s d = 0.45) and modestly lower among students who had completed clinical rotations (12.5 ± 3.2 vs. 13.2 ± 3.5; *t* = 3.56, *p* < 0.001; Cohen’s d = 0.21). Because stigma was included as a secondary contextual construct, it was carried forward descriptively and in correlation analyses rather than in the primary regression models.

### 3.5 Factors associated with PEP knowledge

In bivariate analyses, PEP knowledge scores differed significantly by academic year (F[5, 1,376] = 16.48, *p* < 0.001; partial eta-squared = 0.057), institution (*F*[2, 1,379] = 38.21, *p* < 0.001; partial eta-squared = 0.053), prior PEP training (14.6 ± 3.8 vs. 11.3 ± 4.2; *t* = 12.91, *p* < 0.001; Cohen’s d = 0.81), formal HIV curriculum exposure (12.8 ± 4.1 vs. 10.5 ± 4.3; *t* = 8.62, *p* < 0.001; Cohen’s d = 0.55), and clinical rotation completion (13.4 ± 4.0 vs. 11.2 ± 4.3; *t* = 9.53, *p* < 0.001; Cohen’s d = 0.53). Female students had slightly higher mean knowledge scores than male students (12.4 ± 4.2 vs. 11.7 ± 4.4; *t* = 2.98, *p* = 0.003; Cohen’s d = 0.16). Age (*r* = 0.08, *p* = 0.09), pre-enrollment residence (*t* = 1.56, *p* = 0.12), and prior occupational exposure (*t* = 1.26, *p* = 0.21) were not significantly associated with knowledge.

In the multivariable linear regression model (Table 4), higher knowledge was independently associated with academic year (beta = 0.26, 95% *CI* [0.21, 0.31], *p* < 0.001), prior PEP training (beta = 0.22, 95% *CI* [0.17, 0.27], *p* < 0.001), formal HIV curriculum exposure (beta = 0.12, 95% *CI* [0.06, 0.17], *p* = 0.004), and attendance at Site 1 relative to Site 3 (beta = 0.18, 95% *CI* [0.13, 0.23], *p* < 0.001). The Site 2 versus Site 3 contrast was small and not statistically significant after adjustment. Sex and clinical rotation were not independently associated with knowledge after adjustment. The model explained 19.1% of variance in knowledge score (R-squared = 0.191; *F*[*9,* 1,372] = 35.99, *p* < 0.001). Variance inflation factors were all below 2.0, and regression estimates were materially unchanged in robust-standard-error sensitivity models.

**Table 4:** Multivariable linear regression for PEP knowledge and attitude.

Predictor	Knowledge model beta (95% CI)	Knowledge model *p*	Attitude model beta (95% CI)	Attitude model *p*
Age	0.04 (−0.02, 0.10)	0.18	0.02 (−0.04, 0.08)	0.52
Sex (Female vs. Male)	0.05 (−0.01, 0.11)	0.08	0.07 (0.01, 0.12)	0.017
Academic Year	0.26 (0.21, 0.31)	<0.001	0.04 (−0.01, 0.09)	0.12
Institution (Site 1 vs. Site 3)	0.18 (0.13, 0.23)	<0.001	0.06 (0.01, 0.11)	0.022
Institution (Site 2 vs. Site 3)	0.03 (−0.03, 0.08)	0.34	0.02 (−0.04, 0.07)	0.58
PEP training (Yes vs. No)	0.22 (0.17, 0.27)	<0.001	0.15 (0.09, 0.20)	<0.001
HIV curriculum (Yes vs. No)	0.12 (0.06, 0.17)	0.004	0.04 (−0.02, 0.10)	0.17
Clinical rotation (Yes vs. No)	0.05 (−0.01, 0.11)	0.10	0.09 (0.03, 0.14)	0.005
Knowledge score	—	—	0.28 (0.23, 0.33)	<0.001
R-squared	0.191		0.202	

beta, standardized coefficient; CI, confidence interval. Knowledge Model: F(9, 1,372) = 35.99, *p* < 0.001. Attitude Model: F(10, 1,371) = 34.70, *p* < 0.001. All VIF values below 2.0.

### 3.6 Factors associated with PEP attitude

In bivariate analyses, attitude scores were significantly associated with sex (53.6 ± 8.3 vs. 51.7 ± 8.8; *t* = 4.13, *p* < 0.001; Cohen’s d = 0.22), prior PEP training (55.8 ± 7.5 vs. 51.9 ± 8.7; *t* = 7.48, *p* < 0.001; Cohen’s d = 0.46), clinical rotation completion (54.1 ± 8.1 vs. 51.9 ± 8.8; *t* = 4.62, *p* < 0.001; Cohen’s d = 0.26), and institution (*F*[2, 1,379] = 12.75, *p* < 0.001; partial eta-squared = 0.018). Age (*r* = 0.04, *p* = 0.12) and pre-enrollment residence (*t* = 0.94, *p* = 0.35) were not significantly associated with attitude score.

In the multivariable linear regression model (Table 4), more favorable attitudes were independently associated with higher knowledge score (beta = 0.28, 95% *CI* [0.23, 0.33], *p* < 0.001), prior PEP training (beta = 0.15, 95% *CI* [0.09, 0.20], *p* < 0.001), clinical rotation completion (beta = 0.09, 95% *CI* [0.03, 0.14], *p* = 0.005), female sex (beta = 0.07, 95% *CI* [0.01, 0.12], *p* = 0.017), and attendance at Site 1 relative to Site 3 (beta = 0.06, 95% *CI* [0.01, 0.11], *p* = 0.022). The Site 2 versus Site 3 contrast was not statistically significant. Academic year and formal HIV curriculum exposure were not independently associated with attitude score after covariate adjustment. The model explained 20.2% of variance in attitude score (R-squared = 0.202; *F*[*10,* 1,371] = 34.70, *p* < 0.001).

### 3.7 Institutional differences

PEP knowledge scores differed significantly across institutions in unadjusted analyses: Site 1, 13.2 ± 3.9; Site 2, 11.6 ± 4.3; and Site 3, 10.9 ± 4.4 (*F*[2, 1,379] = 38.21, *p* < 0.001). After adjustment for age, sex, and academic year, the institutional difference remained significant (ANCOVA: *F*[2, 1,376] = 18.67, *p* < 0.001; partial eta-squared = 0.026), with adjusted means shown in Table 5.

**Table 5:** PEP knowledge and attitude by institution (adjusted).

Outcome	Site 1 M (95% CI)	Site 2 M (95% *CI*)	Site 3 M (95% *CI*)	*F (df)*	*p*
Knowledge	13.3 (12.6–14.0)	11.4 (10.7–12.1)	10.9 (10.2–11.6)	18.67 (2, 1,376)	< 0.001
Attitude	53.4 (52.3–54.5)	52.4 (51.2–53.6)	52.0 (50.8–53.2)	2.77 (2, 1,376)	0.063

ANCOVA adjusted for age, sex, and academic year. M, estimated marginal mean; CI, confidence interval.

**Figure 1:**
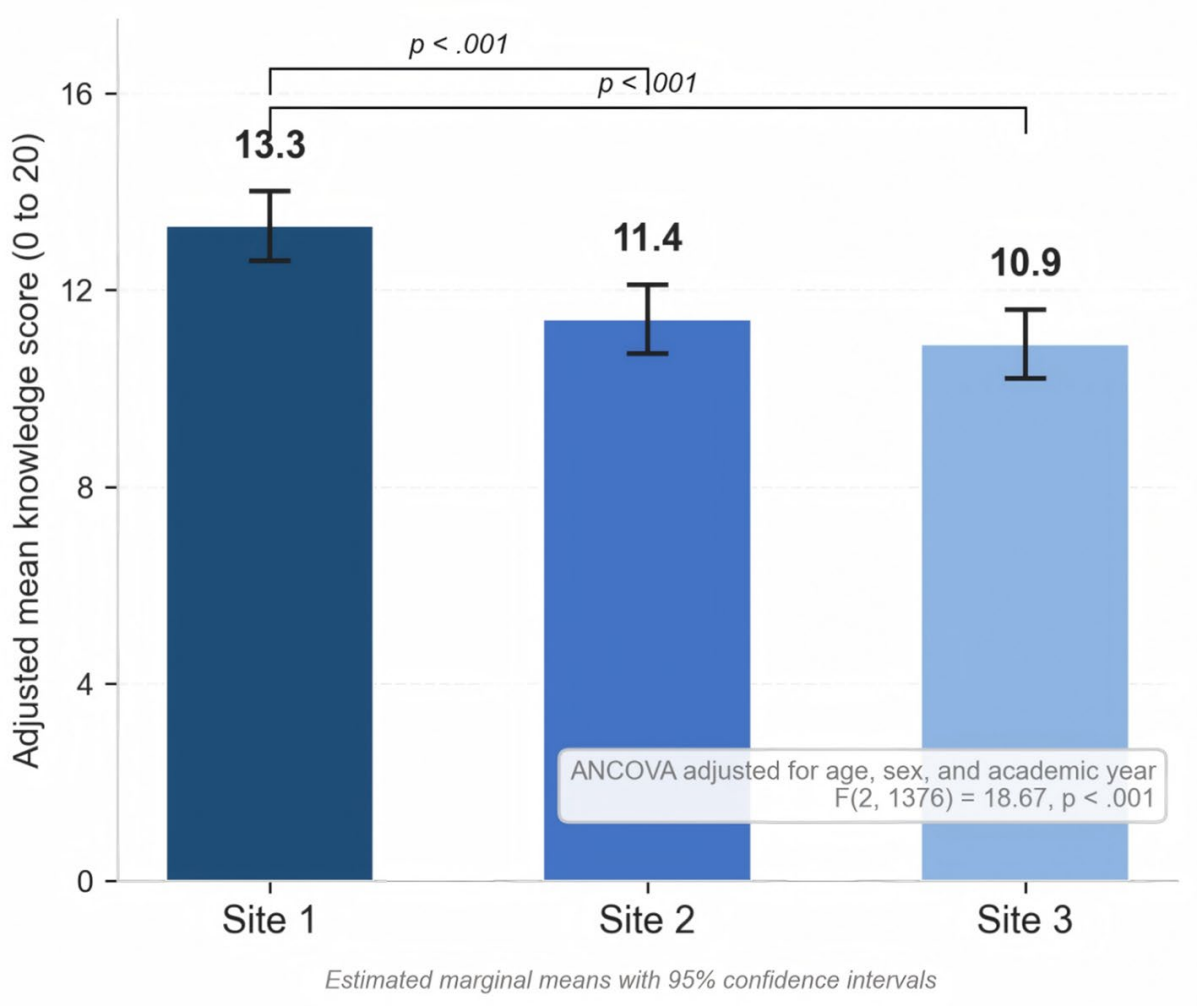
Adjusted mean HIV post-exposure prophylaxis knowledge scores by institution. Bars show estimated marginal means for total PEP knowledge score after adjustment for age, sex, and academic year in analysis of covariance. Error bars represent 95% confidence intervals. Adjusted mean knowledge scores were 13.3 (95% CI 12.6–14.0) at Site 1, 11.4 (95% CI 10.7–12.1) at Site 2, and 10.9 (95% CI 10.2–11.6) at Site 3. The overall adjusted institutional effect was statistically significant (ANCOVA: F[2, 1,376] = 18.67, *p* < 0.001; partial eta-squared = 0.026). Bonferroni-adjusted pairwise comparisons showed higher adjusted knowledge scores at Site 1 than at Site 2 and Site 3 (both *p* < 0.001), whereas Site 2 and Site 3 did not differ significantly after adjustment (*p* = 0.29). Knowledge score ranged from 0 to 20, with higher scores indicating greater HIV PEP knowledge.

Bonferroni-adjusted pairwise comparisons showed higher adjusted knowledge scores at Site 1 than at Site 2 and Site 3 (both *p* < 0.001), whereas Site 2 and Site 3 did not differ significantly after adjustment (*p* = 0.29). Adjusted institutional differences in knowledge score are shown in Figure 1.

**Figure 2:**
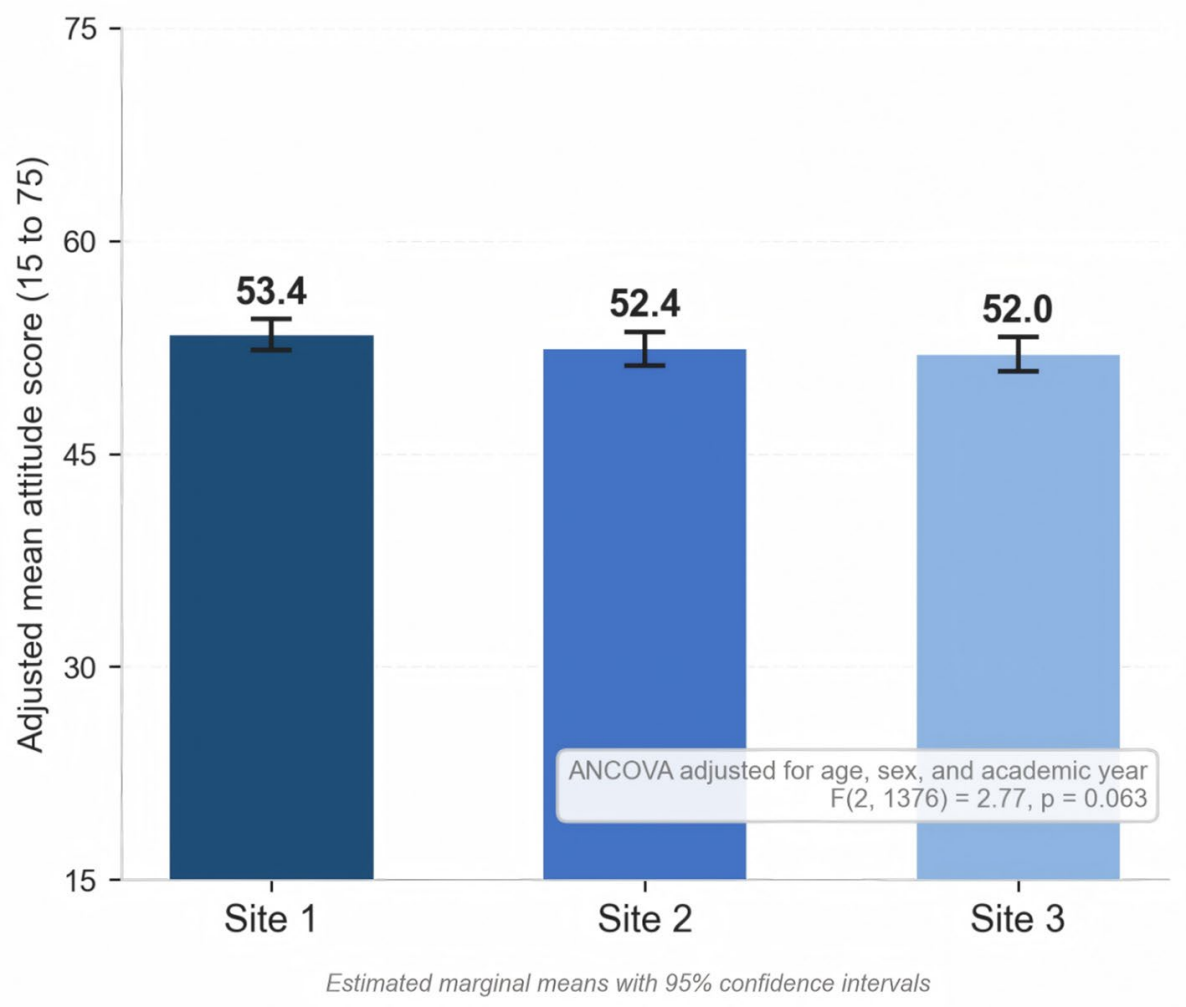
Adjusted mean HIV post-exposure prophylaxis attitude scores by institution. Bars show estimated marginal means for total PEP attitude score after adjustment for age, sex, and academic year in analysis of covariance. Error bars represent 95% confidence intervals. Adjusted mean attitude scores were 53.4 (95% CI 52.3–54.5) at Site 1, 52.4 (95% CI 51.2–53.6) at Site 2, and 52.0 (95% CI 50.8–53.2) at Site 3. The adjusted institutional effect was small and did not meet the conventional 0.05 threshold for statistical significance (ANCOVA: F[2, 1,376] = 2.77, *p* = 0.063; partial eta-squared = 0.004). Attitude score ranged from 15 to 75, with higher scores indicating more favorable attitudes toward HIV PEP. The scale included items addressing perceived importance of PEP, willingness to seek or recommend PEP, support for inclusion of PEP in medical curricula, confidence in communication about PEP, and perceived barriers such as accessibility, cost, and confidentiality.

Attitude scores were 53.3 ± 8.3 at Site 1, 52.5 ± 8.8 at Site 2, and 52.0 ± 8.9 at Site 3 in the corresponding unadjusted summaries. The adjusted site comparison for attitudes remained small and did not reach the conventional 0.05 threshold (ANCOVA: *F*[2, 1,376] = 2.77, *p* = 0.063; partial eta-squared = 0.004). The site pattern for attitudes should therefore be interpreted cautiously. Adjusted institutional differences in attitude score are shown in Figure 2.

### 3.8 Relationship of knowledge, attitude, and stigma

PEP knowledge and attitude scores were positively correlated (*r* = 0.29, 95% *CI* [0.24, 0.34], *p* < 0.001). The magnitude of the correlation was similar across institutions, with site-specific coefficients ranging from 0.27 to 0.31. HIV-related stigma was negatively correlated with knowledge (*r* = −0.15, *p* < 0.001) and with attitude (*r* = −0.24, *p* < 0.001). The stigma correlations were modest in magnitude but directionally consistent with the broader pattern linking lower stigma to stronger PEP preparedness.

### 3.9 Sensitivity analyses

**Figure 3:**
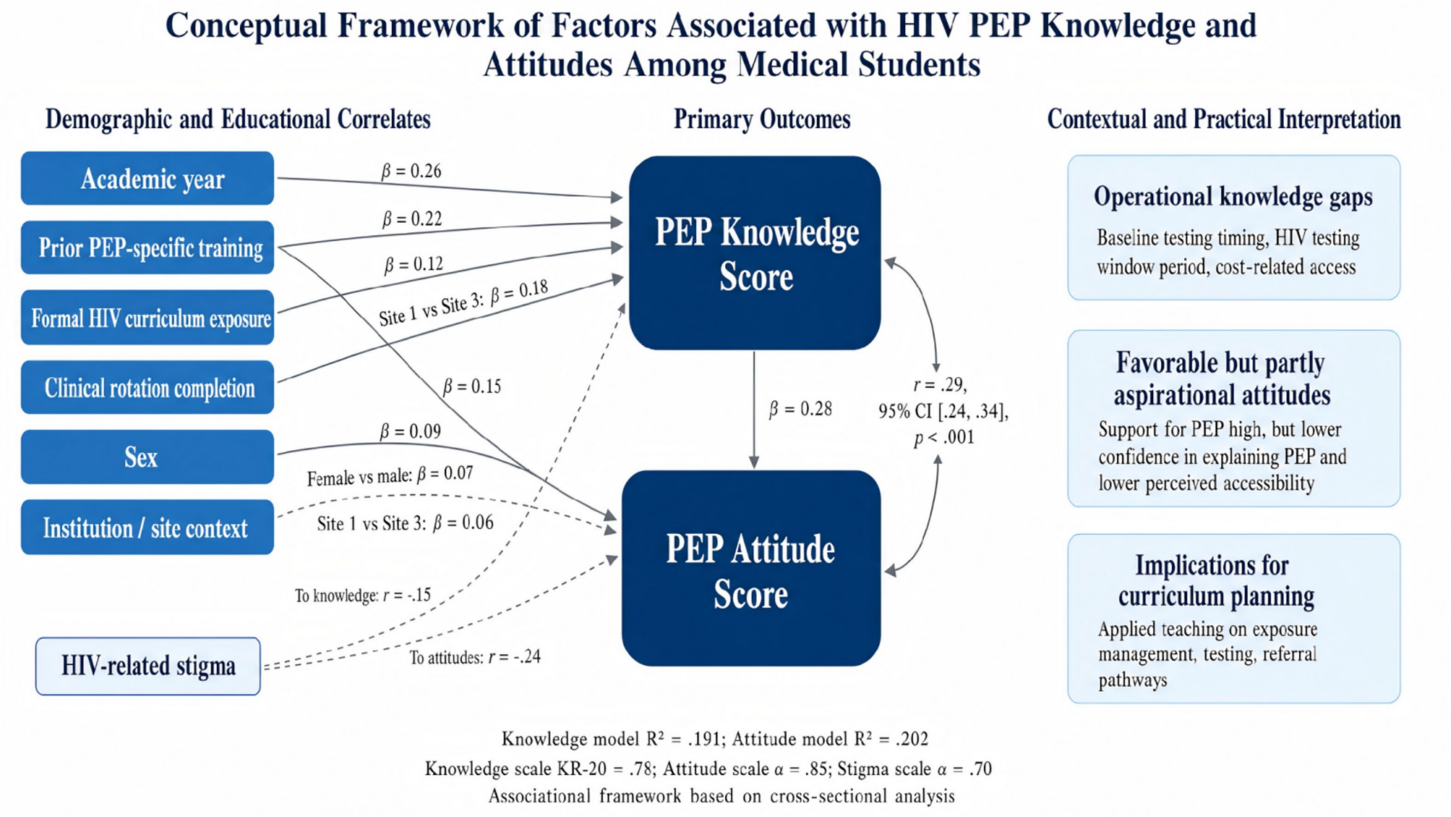
Conceptual framework of demographic, educational, and institutional correlates of HIV post-exposure prophylaxis knowledge and attitudes among medical students. The framework summarizes the main variables examined in this multi-center cross-sectional survey of 1,382 medical students from three Chinese medical schools. The primary outcomes were total PEP knowledge score (0–20) and total PEP attitude score (15–75). Academic year, prior PEP-specific training, formal HIV curriculum exposure, clinical rotation completion, sex, and institutional context were evaluated as correlates of outcome variation. In the multivariable models, higher knowledge score was associated with academic year (beta = 0.26), prior PEP training (beta = 0.22), HIV curriculum exposure (beta = 0.12), and attendance at Site 1 relative to Site 3 (beta = 0.18). More favorable attitude score was associated with higher knowledge score (beta = 0.28), prior PEP training (beta = 0.15), clinical rotation completion (beta = 0.09), female sex (beta = 0.07), and attendance at Site 1 relative to Site 3 (beta = 0.06). Knowledge and attitude scores were positively correlated (*r* = 0.29, 95% CI [0.24, 0.34], *p* < 0.001). HIV-related stigma showed negative correlations with both knowledge (*r* = −0.15) and attitudes (*r* = −0.24). Scale reliability estimates were KR-20 = 0.78 for knowledge, Cronbach’s alpha = 0.85 for attitudes, and Cronbach’s alpha = 0.70 for stigma.

Sensitivity analyses excluding participants who failed two or more attention checks (*n* = 79), participants with response times below 240 s (*n* = 64), and participants who self-rated their response honesty as untruthful (*n* = 23) yielded the same direction and similar magnitude for all primary associations. Across those alternative analytic sets, the absolute change in the main standardized coefficients was less than 0.02, and no primary conclusion changed. Academic year, prior PEP training, and HIV curriculum exposure remained associated with higher knowledge, and knowledge score, prior PEP training, and clinical rotation remained associated with more favorable attitudes. Figure 3 provides an integrated summary of the observed association structure across the primary variables.

## 4 Discussion

Knowledge data revealed meaningful compartmentalization between domains of understanding. Students performed adequately on items requiring basic factual recognition of PEP as a preventive strategy, yet accuracy dropped sharply on questions demanding applied operational reasoning, specifically, whether to delay PEP until baseline HIV test results were available, the implications of the HIV-testing window period, and the practical steps for accessing PEP services. This pattern suggests that classroom- or lecture-based exposure to PEP as a concept may not be sufficient to equip students with the procedural knowledge needed to manage a real-world exposure scenario. The finding aligns with observations from Italy, where less than half of surveyed healthcare professionals correctly identified the preventive benefits of PEP and substantial proportions were unaware of workplace PEP protocols (16). Similarly, studies among healthcare workers in Southern Africa and Nigeria have documented that while general awareness of PEP is often high, detailed knowledge encompassing correct timing, drug regimens, and stepwise clinical management remains fragmented (17, 18). In a cohort of clinical students in northern Nigeria, although 98% had heard of PEP, only 26% demonstrated adequate knowledge (18).

The present findings also extend prior work that has examined HIV knowledge and attitudes among health-science students globally. Much of that literature has focused on general HIV/AIDS knowledge rather than PEP-specific comprehension and beliefs (15, 19, 20). Investigations that have addressed PEP specifically were often conducted in Western or Middle Eastern settings, employed single-center designs, or recruited small and homogeneous samples (10, 12, 14). In China, prior studies have shown low awareness or use of PEP among key populations and college students, including young men who have sex with men and broader university student samples (6, 21–23, 40, 41). The current study therefore contributes by describing PEP-specific knowledge and attitudes across three participating medical schools, while also showing why the findings should be interpreted as institution-specific evidence rather than as national estimates.

Despite a generally positive attitudinal orientation toward PEP, reflected in a mean score of 52.8 on a 15–75 scale, the data reveal a more nuanced pattern when examined at the item level. Respondents endorsed the importance of PEP as a prevention tool and expressed willingness to recommend it to patients, yet these favorable dispositions coexisted with markedly lower confidence in their own ability to initiate PEP counseling or navigate the clinical steps required for timely administration. This divergence between abstract support and practical assurance mirrors findings among medical trainees internationally. A survey of graduate medical trainees in New York City found that most endorsed the importance of providing HIV prevention services, yet knowledge of nPEP protocols remained poor, and self-reported comfort with prescribing was low even among those who considered prevention part of their role (24). Evidence from doxycycline-based PEP is not directly equivalent to HIV PEP because the target pathogens, indications, and prescribing considerations differ. However, it illustrates a broader problem in STI-prevention education: even when clinicians recognize the concept of post-exposure prophylaxis, they may remain uncertain about pathogen-specific efficacy, eligibility, and real-world prescribing. Among Italian dermatologists managing sexually transmitted infections, awareness of doxycycline-based PEP was universal, yet confidence in its efficacy against specific pathogens varied considerably, and fewer than one in five had actually prescribed it (25). The gap between endorsement and operational confidence in the present sample thus aligns with a broader literature in which favorable attitudes toward prevention do not automatically translate into perceived self-efficacy for clinical use.

Stigma emerged as a negative correlate of both knowledge and attitudes in the present study, adding another layer of complexity to the attitude pattern. Students who endorsed stigmatizing beliefs toward people living with HIV reported less favorable attitudes toward PEP, a finding consistent with studies showing that HIV stigma undermines engagement with prevention services among both patients and providers (26, 39). In Indonesia, nursing and medical students with higher HIV stigma scores demonstrated lower knowledge levels, and the relationship persisted after adjustment for demographic and educational factors. Within healthcare settings, stigma manifests not only as overt discrimination but also as subtle avoidance behaviors; nurses in Bandung who reported good HIV knowledge nonetheless expressed reluctance to care for HIV patients due to fear of transmission, revealing a separation between cognitive understanding and affective response (27). The present data extend this observation by showing that stigma correlates with attitudes toward a prevention tool itself, not merely attitudes toward affected populations. This suggests that stigma reduction may need to accompany technical PEP education when preparing trainees for patient-centered risk communication.

Prior PEP-specific training showed a stronger association with higher knowledge scores than general HIV curriculum exposure, a pattern consistent with prior literature. Among clinical students in northern Nigeria, previous PEP training independently predicted adequate knowledge, with those lacking such training having significantly lower odds of satisfactory scores (aOR 0.43, 95% CI 0.23 to 0.80) (18). Similarly, Polish secondary school students who received a single educational session on HIV demonstrated measurable improvement in PEP-related knowledge 1 year later, though gaps persisted in applied domains (28). The distinction between dedicated PEP training and broader HIV curriculum exposure may reflect differences in content depth and pedagogical focus. General HIV curricula often emphasize epidemiological and transmission knowledge, whereas PEP-specific training tends to address procedural steps, timing, and clinical decision-making, precisely the applied domains where knowledge deficits were concentrated in the present sample. This interpretation aligns with findings from a quasi-experimental study among dental students, where a structured educational intervention significantly improved knowledge of post-exposure prophylaxis procedures, even as gaps in general infection control knowledge remained (29).

Institutional differences observed in this study are consistent with patterns reported across diverse settings, although the cross-sectional design does not allow the differences to be attributed causally to site characteristics. Students at Site 1, a nationally prominent medical school with more visible tertiary clinical resources and optional HIV/STI-related seminars, demonstrated significantly higher PEP knowledge and slightly more favorable attitudes than those at Site 3. This distinction persisted after adjustment for individual-level factors, suggesting that institutional context may be associated with learners’ readiness. In a survey of primary care providers across six high-HIV-burden U.S. jurisdictions, those practicing in academically affiliated or resource-rich settings reported greater familiarity with nPEP (aPR 1.32) and higher rates of PrEP prescribing compared with providers in community-based or less-resourced practices (30). Similarly, among healthcare workers in Tanzania, those employed at facilities with established PEP services had significantly higher odds of adequate knowledge (AOR 3.9) (31), and in Ethiopia, prior training availability at the institutional level emerged as a strong correlate of knowledge adequacy (32). These findings collectively suggest that educational environment, specialized curricula, visible clinical protocols, and institutional emphasis on HIV prevention may be relevant structural correlates of PEP competence.

### 4.1 Limitations

Several methodological considerations temper the interpretation of these findings. The convenience sampling strategy, which relied on WeChat-based recruitment within three medical schools, introduces selection bias and limits the generalizability of results to the broader population of Chinese medical students. Students who chose to participate may differ systematically from non-participants in their baseline interest in HIV prevention, academic engagement, access to institutional information channels, or comfort discussing HIV-related topics, potentially inflating the observed knowledge and attitude scores. Because recruitment was conducted through group messages rather than a defined roster, the denominator of invited or exposed students was unknown and a response rate could not be calculated. Recruitment through advisor-linked groups may also have generated subtle response pressure, even though the survey was anonymous and voluntary. In addition, the study did not collect information on participants’ personal sexual health history, previous STI clinic attendance, prior HIV testing, PrEP or nPEP use, or personal exposure concerns. These unmeasured experiences may have shaped both knowledge and attitudes and therefore represent a possible source of residual bias. Finally, the participating schools differed in size, teaching-hospital resources, and likely exposure to HIV/STI services; these site descriptors help interpretation but do not establish representativeness.

The study-specific instrument, while adapted from established frameworks identified in systematic reviews of HIV KAP tools (33, 34), has not undergone formal validation beyond expert content review, pilot testing, internal consistency assessment, and pragmatic factor-structure checks. The content validity indices, KR-20, Cronbach’s alpha values, item-total correlations, and confirmatory factor analysis results support basic measurement adequacy for this survey, but they do not establish criterion validity, test–retest reliability, measurement invariance across institutions, or external validity in other medical student populations. The decision to code “unsure” responses as incorrect may have modestly underestimated students’ partial knowledge, particularly in clinically nuanced domains such as the HIV testing window period or the appropriate timing of PEP initiation (35). Self-reported measures of HIV curriculum exposure and prior PEP training are also vulnerable to recall inaccuracy, and the cross-sectional design precludes any assessment of knowledge retention, attitudinal stability, or causal direction over time. As noted in recent reviews, cross-sectional KAP surveys provide a useful snapshot but cannot capture the dynamic interplay between training, clinical experience, and competency development (33, 36).

### 4.2 Implications

The knowledge pattern observed in this study, stronger factual recall paired with weaker applied operational understanding, directs attention toward curricular design that moves beyond didactic HIV content. While general HIV curriculum exposure was associated with knowledge, the stronger association of prior PEP-specific training with both knowledge scores and attitudes suggests that dedicated, skills-oriented modules may be useful additions to broad survey courses. Integrating case-based exercises that involve timing of PEP initiation, interpretation of window-period testing, confidentiality-sensitive counseling, referral pathways, and navigation of cost-related barriers could bridge the gap between declarative knowledge and clinical readiness (29, 37). Limited provider knowledge may also affect patient-level access: future clinicians who are uncertain about indications, urgency, or follow-up requirements may provide incomplete risk communication, delay referral, or fail to correct misconceptions about HIV transmission and prevention. Such gaps could hinder patients’ proper access to timely PEP and may inadvertently reinforce high-risk behaviors if risk factors and transmission dynamics are poorly explained. Brief educational interventions have been shown to improve PEP knowledge among students and community populations (28, 38), and similar approaches could be embedded within existing infectious disease or community health rotations. The parallel finding that confidence in PEP provision was relatively lower, despite favorable attitudes, reinforces the need for practice-oriented teaching, such as standardized patient encounters or simulation sessions, rather than lecture alone.

## 5 Conclusion

Among medical students at three participating Chinese medical schools, PEP knowledge was moderate overall but weaker for applied operational details than for basic factual content. Attitudes toward PEP were more favorable than knowledge levels alone would suggest, although uncertainty about accessibility and communication remained evident. Prior PEP-specific training, academic year, and institutional site were associated with score variation, but these cross-sectional associations should not be interpreted causally. The findings support a practical curricular response in the participating settings, including more explicit teaching on exposure assessment, immediate post-exposure management, baseline and follow-up testing, patient-centered risk communication, and local access pathways. At the same time, convenience sampling, self-reported outcomes, and limited psychometric validation of the study-specific questionnaire restrict the scope of inference.

## Data Availability

The raw data supporting the conclusions of this article will be made available by the authors, without undue reservation.

## Footnotes

1
https://www.wjx.cn
